# What Factors Are Associated with Adolescents' School Break Time Physical Activity and Sedentary Time?

**DOI:** 10.1371/journal.pone.0056838

**Published:** 2013-02-13

**Authors:** Nicola D. Ridgers, Anna Timperio, David Crawford, Jo Salmon

**Affiliations:** Centre for Physical Activity and Nutrition Research (C-PAN), Deakin University, Melbourne, Australia; Pennington Biomedical Research Center, United States of America

## Abstract

**Purpose:**

Adolescents' physical activity levels during school break time are low and understanding correlates of physical activity and sedentary time in this context is important. This study investigated cross-sectional and longitudinal associations between a range of individual, behavioural, social and policy/organisational correlates and objectively measured school break time physical activity and sedentary time.

**Methods:**

In 2006, 146 adolescents (50% males; mean age = 14.1±0.6 years) completed a questionnaire and wore an accelerometer for ≥3 school days. Time spent engaged in sedentary, light (LPA) and moderate-to-vigorous physical activity (MVPA) during school break times (recess and lunchtime) were calculated using existing cut-points. Measures were repeated in 2008 among 111 adolescents. Multilevel models examined cross-sectional and longitudinal associations.

**Results:**

Bringing in equipment was cross-sectionally associated with 3.2% more MVPA during break times. Females engaged in 5.1% more sedentary time than males, whilst older adolescents engaged in less MVPA than younger adolescents. Few longitudinal associations were observed. Adolescents who brought sports equipment to school engaged in 7.2% less LPA during break times two years later compared to those who did not bring equipment to school.

**Conclusion:**

These data suggest that providing equipment and reducing restrictions on bringing in sports equipment to school may promote physical activity during school recess. Strategies targeting females' and older adolescents', in particular, are warranted.

## Introduction

Regular physical activity during adolescence is important for physical and mental health [1], [2]. There is widespread concern, however, that adolescents are not sufficiently active to benefit health. Guidelines for physical activity recommend that youth should engage in at least 60 minutes of moderate-to-vigorous intensity physical activity every day [3], [4], [5], yet in Australia only 25% of males and 13% of females aged 14–16 years meet these recommendations [6]. This is concerning as physical activity levels tend to decline further across adolescence [7]. The promotion of physical activity to adolescents is therefore critical.

Adolescents spend a significant proportion of their weekdays in the school environment, which provides an opportunity to develop and implement interventions that can reach a substantial proportion of the adolescent population on a daily basis. During the school day, one opportunity for adolescents to engage in physically active behaviours is through school break times (recess and lunchtime), which is a mandatory part of the school day in many countries [8]. Whilst school break times can account for up to 20% of the school day, little research has investigated the physical activity levels of adolescents during this time. Of the research conducted in this context, 41.5% of males and 32.6% of female's self-report daily participation in physical activity during break time [9], though decreasing levels of physical activity have been reported across school grades [10] and over time [11]. Despite these decreases, school break times have still been found to contribute up to 23% of adolescents' daily moderate-to-vigorous physical activity [11]. This highlights the potential for break time interventions to not only increase physical activity levels in this context, but to also benefit daily physical activity levels.

To effectively intervene it is important to understand factors that influence physical activity levels and sedentary time at school [12], particularly as these factors may vary according to the context [9], [13]. Social-ecological models provide a useful framework for understanding physical activity and sedentary time [14] as they are complex behaviours that are influenced by a number of factors at multiple levels [15], [16]. These models suggest that individual, social, physical environmental and policy/organisational factors interact to promote or restrict participation in physical activity [14]. Some cross-sectional research into correlates of physical activity during school break time in adolescents has identified social [17], physical environmental [9], [10] and policy [18] influences on self-reported physical activity participation. However, no studies to date have used objective measures or examined correlates of adolescent sedentary time or light physical activity during break time, despite light intensity physical activity accounting for approximately 50% of adolescent's activity levels [11]. It is possible that correlates may differ for different intensities of physical activity during break times [19], which is an important consideration for the development of interventions aiming to increase physical activity and decrease sedentary time, especially as evidence regarding the effectiveness of school-based interventions on adolescent physical activity is inconclusive [20].

Given the paucity of research examining correlates of adolescents' break time physical activity and sedentary behaviour [21], there is a need to identify which variables could be modified to inform the development of effective interventions in this population. Moreover, as factors that influence changes in break time physical activity are poorly understood, research is needed to examine whether cross-sectional correlates explain changes over time. Identifying cross-sectional and longitudinal correlates of break time activity will have the potential to influence the activity levels of adolescents on a daily basis by informing future policy and practice.

The aim of this study was to examine cross-sectional and longitudinal associations between a range of individual, behavioural, social and policy/organisational factors and objectively measured school break time physical activity and sedentary time in adolescents.

## Methods

### Ethics statement

Data were drawn from the first (2006; T2) and second (2008; T3) follow-ups of adolescents who participated in the Health, Eating and Play Study (HEAPS) in 2002/3. Ethical approval was provided by the Deakin University Human Research Ethics Committee, the Department of Education and Training Victoria, and the Victorian Catholic Education Office. Informed written consent was obtained from parents and secondary school children at each of the follow-up data collections.

### Sample

Stratified random sampling proportionate to school size (enrolment greater than 200 pupils) was employed to recruit schools from metropolitan Melbourne, Australia, into the study. At baseline (2002/2003; T1), 24 (9 high, 7 middle, and 8 low socioeconomic status) elementary (primary) schools agreed to participate in the study. All children in Grades 5–6 (aged 10–12 years) were invited to participate, and 947 children (434 males, 513 females; mean age  = 11.2±0.6 years) returned parental consent forms. In Australia it is an ethics requirement for parents to provide active informed consent on behalf of themselves and their child; thus no information is available concerning non-responders at baseline. Families were asked whether they could be contacted for further research and those that agreed (n = 474) were re-contacted and subsequently invited to participate in the 2006 follow-up data collection. Of those invited, 200 families participated in the first follow-up (T2), and 145 families participated in the second follow-up (T3) [22].

### Physical activity measure

Adolescent physical activity was measured for 60 seconds over eight consecutive days using a hip-mounted uni-axial accelerometer (7164 ActiGraph, Fort Walton Beach, Florida, USA). Adolescents were instructed to wear the accelerometer during all waking hours except during water-based activities (e.g. swimming, bathing), and were provided with information concerning the correct wear and care of the monitor. Accelerometers have been validated against doubly labelled water and indirect calorimetry in adolescents in laboratory and free-living contexts [23].

Accelerometer data were downloaded using ActiGraph software. School bell times for each school were used to analyse recess and lunchtime data using a customised Excel macro. Non-wear time was defined as sustained 20-minute periods of zero counts. Adolescents who produced counts for at least 50% of recess and lunchtime on at least 3 schooldays were retained for further analyses.

Data were analysed using age-specific cut-points [23] to obtain time spent in light (LPA; 1.5–3.9 METs), moderate (MPA; 4–5.99 METs) and vigorous (VPA; ≥6 METs) intensity physical activity. A threshold of 4 METs was chosen to represent MPA as brisk walking has been associated with an energy cost of 4 METs in calibration studies [24]. Sedentary time was defined as <100 counts·min^−1^ [25]. MPA and VPA were summed to obtain moderate-to-vigorous physical activity (MVPA). The duration of time adolescents engaged in sedentary, LPA and MVPA during recess and lunchtime were determined for days that met the minimum inclusion criteria of counts recorded for ≥50% of break time. Recess and lunchtime physical activity data were summed to provide the total time adolescents engaged in sedentary, LPA and MVPA during daily school break time, and averaged per valid day. The percentage of time adolescents engaged in sedentary (%SED), light (%LPA), and MVPA (%MVPA) was determined by dividing the time spent in each of these intensities by total available time during breaks. To adjust for the effect of daily physical activity levels on break time physical activity, the total time that adolescent's spent in engaged in sedentary time, LPA and MVPA per day at each time point was determined. Days that met the inclusion criteria for a valid day were identified, and the average time spent in sedentary time, LPA and MVPA per day was calculated and used in the analyses.

### Correlates

All adolescents and parents completed a self-report questionnaire at each time point. Eleven variables were analysed as potential correlates of adolescent break time physical activity and sedentary time using the social-ecological framework [14].

#### Individual variables

Parents completed items requesting demographic information about the adolescent (e.g. age, date of birth, school level) and the family as a whole (e.g. marital status, maternal education, maternal employment status). The self-reported highest level of maternal education was used as a proxy-measure of socio-economic status (SES) and was classed as low (some high school attendance or less), medium (high school or trade certificate completed) and high (tertiary education) [26].

Adolescents' *s*tature (m) and body mass (kg) were measured at each time point without shoes by trained data collectors using a portable stadiometer and digital scales. Body mass index (BMI; kg/m^2^) was calculated, and adolescents were classified as non-overweight or overweight/obese using BMI cut-off points developed by the International Obesity Taskforce [27].

Perceived personal barriers to physical activity were assessed by adolescents' reported agreement with nine statements on a 5-point Likert type scale from strongly disagree' (1) to ‘strongly agree’ (5). Example statements included; I don't like physical activity; I'm not the sport type; and I look funny when I am physically active. Responses to the statements were summed and averaged (Cronbach's α = 0.81); a high score reflects a higher barrier.

#### Behavioural variables

To examine behaviour during break time, adolescents were asked how often they engaged in sport and/or physical activity during break time, brought sports equipment to school for use during break time or borrowed sports equipment from school for use during break time on a 5-point Likert-type scale (1 = everyday, 2 = most days, 3 = sometimes, 4 = hardly ever, 5 = never). These variables were dichotomised into ‘yes’ (i.e. engaged in this behaviour most days) and ‘no’ [9], [17].

#### Social variables

Social support was determined using responses to six questions asking how often they had been encouraged 1) by their friends and 2) by their family to do more physical activity, walk to school and other places, and play more sport. Adolescents responded on a 3-point scale (1 = Never, 2 = Sometimes, 3 = Often) which was summed and averaged to provide an overall indicator of peer (Cronbach's α = 0.69) and family support (Cronbach's α = 0.76). A higher score reflected greater support.

#### Policy/organisational variable

School bell times were recorded by a contact within each of the schools and were used to determine the length of recess and lunch breaks. The durations of recess and lunch were summed to provide a total break time duration in minutes.

### Statistical analyses

Chi-square analyses were initially conducted on descriptive data to examine differences between adolescents who participated at follow-up (n = 183) to adolescents who participated at baseline (T1; n = 764) only. Cross-sectional and longitudinal analyses were performed using multilevel models. Multilevel models are the most appropriate data analysis technique for nested data [28]. A two-level structure was used, with adolescents defined as the first level and secondary school defined as the second level.

The development of the multivariate analyses consisted of two steps. Firstly, a crude model was constructed to identify predictor variables that were significantly associated with the outcome variable (*p*<0.1) [29]. Regression coefficients were assessed for significance using the Wald statistic. All predictor variables associated with the outcome variable were entered into the fully adjusted model. Before entry into the adjusted model, the correlation between predictor variables was determined. Correlations coefficients that were greater than r = 0.5 were excluded from the adjusted model as a more conservative approach than suggested (r = 0.7) [30]. The cross-sectional analyses included all T2 variables associated with the outcome variable at T2. The longitudinal analyses included all T2 variables associated with the outcome at T3, adjusted for T2 physical activity or sedentary time. Separate analyses were conducted for%SED,%LPA and%MVPA. All analyses were conducted using MLwiN 1.10 software (Institute of Education, University of London, UK).

## Results

### Sample

Compared to those who participated at baseline (T1) only, a higher proportion of adolescents who participated at follow-up were of higher SES (46% vs. 19%) based on maternal education. No differences were observed for maternal employment or marital status. Complete cross-sectional data at T2 were collected from 146 adolescents (73 males, 73 females; mean age = 14.1±0.6 years). Eighty-four per cent of adolescents had carers who were married. Approximately one-third (34%) of mothers reported full-time employment, while 36% reported part-time employment. Based on maternal education, 42% were of medium SES and 38% were of high SES. The longitudinal sample comprised of 111 adolescents (56 males, 55 females; mean age = 15.6±0.7 years). There were no significant differences in physical activity levels, maternal employment and marital status between those with longitudinal data and those without, although adolescents with follow-up data at T3 had mothers with significantly higher education (43% vs. 32%). The average total daily break time duration was 86.4 (±6.5) minutes at T2 and 86.5 (±6.8) minutes at T3. Table 1 reports break time physical activity levels at T2 and at T3 and the change in physical activity levels in the longitudinal sample. Descriptive information concerning individual, behavioural, social and policy/organisational variables is presented in Table 2.

**Table 1:** Physical activity during school break time at T2 and T3 (raw data).

	Cross-sectional sample (n = 146)	Longitudinal sample (n = 111)	
	Mean (SD)	Mean (SD)	Change (T2 to T3)
%SED	52.9 (12.5)	49.7 (15.4)	−3.3 (13.7)
%LPA	39.4 (9.6)	43.1 (11.9)	3.6 (10.2)
%MVPA	7.6 (5.7)	7.2 (7.1)	0.3 (8.1)

**Table 2:** Information concerning variables used in the analyses (mean (SD) unless stated).

	Range	T2 Whole sample (n = 146)
**Individual factors**		
Boys (%)	N/A	50
Body mass index (kg/m^2^)	14.9–36.4	21.1 (3.4)
Overweight (%)	N/A	19.9
Barriers to physical activity	1–5	1.9 (0.6)
**Behavioural factors**		
Engage in physical activity during recess (%)^1^	Yes/No	37.9
Bring in sports equipment (%)^1^	Yes/No	10.3
Borrow sports equipment (%)^1^	Yes/No	12.4
**Social factors**		
Family support	1–5	1.5 (0.5)
Peer support	1–5	1.9 (0.7)
**Organisational/policy factors**		
Break time duration (min)	65–90	86.4 (6.5)

1 Percentage of adolescents reporting ‘yes’.

### Cross-sectional results

A significant cross-sectional association was found between sedentary time and sex. Females spent a greater proportion of school break time sedentary compared to males (Table 3). Negative associations were observed for sex and high maternal education with%LPA during break time at T2, with females and adolescents whose mother had a tertiary education engaging in less%LPA (Table 4). Females and adolescents in higher year levels at T2 engaged in less%MVPA, and those who brought in sports equipment to school engaged in more%MVPA at T2 compared to other adolescents (Table 5).

**Table 3:** Cross-sectional and longitudinal associations between individual, behavioural, social and organisational/policy factors and break time%sedentary time.

Variables	Cross-sectional		Longitudinal	
	Crude model^a^	Fully-adjusted model^b^	Crude model^a^	Fully-adjusted model^c^
	β (95% CI)	β (95% CI)	β (95% CI)	β (95% CI)
**Individual factors**				
Daily SED time	0.06 (0.04, 0.08)^***^	0.05 (0.02, 0.07)^***^	0.05 (−0.07, 0.06)	
Sex^1^	8.6 (5.1, 12.1)^***^	5.1 (1.4, 8.8)^***^	12.2 (6.3, 18.2)^***^	10.4 (4.1, 16.6)^***^
Maternal education				
Low	Ref	–	Ref	–
Medium	−1.7 (−5.1, 1.8)	–	3.0 (−2.7, 8.8)	–
High	1.8 (−1.9, 5.5)	–	−5.0 (−0.9, 10.9)*	−4.0 (−9.4, 1.4)
School year	4.9 (1.1, 8.7)^***^	2.6 (−0.9, 6.1)	5.1 (−0.7, 10.9)*	5.4 (0.0, 10.7)^**^
BMI Category				
Normal weight	Ref	–	Ref	–
Overweight	−3.6 (−8.5, 1.3)	–	−2.7 (−10.2, 4.8)	–
Barriers to PA	−0.3 (−3.5, 3.0)	–	1.5 (−3.0, 4.5)	–
**Behavioural factors**				
Sport/PA during recess	−6.3 (−10.1, −2.5)^***^	0.1 (−4.1, 4.4)	−9.1 (−15.3 −2.9)^***^	−2.3 (−9.2, 4.6)
Bring sports equipment	−5.9 (−11.6, −0.2)^**^	−4.2 (−9.6, 1.2)	−10.2 (−18.6, −1.8)*	−6.3 (−15.2, 2.6)
Borrow sports equipment	−6.4 (−12.3, −0.6)^**^	−2.3 (−8.0, 3.3)	−5.6 (−14.2, 3.0)	–
**Social factors**				
Peer support	1.5 (−2.3 to 5.2)	–	0.6 (−5.6, 6.8)	–
Family support	1.5 (−1.1, 4.1)	–	1.8 (−2.8, 6.3)	–
**Organisational/policy factors**				
Break time duration	0.1 (−0.3, 0.4)	–	−0.1 (−0.5, 0.4)	–
**Total variance explained**		31.8%		21.0%

* *p*<0.1, ^**^ *p*<0.05, ^***^ *p*<0.01.

–  =  Not entered in fully-adjusted model.

1 Males are the referent group.

a Separate models for each dependent variable.

b Adjusted for all significant variables from the crude model.

c Adjusted for T2 sedentary time and all significant variables from the crude model.

**Table 4:** Cross-sectional and longitudinal associations between individual, behavioural, social and organisational/policy factors and break time%LPA.

Variables	Cross-sectional		Longitudinal	
	Crude model^a^	Fully-adjusted model^b^	Crude model^a^	Fully-adjusted model^b^
	β (95% CI)	β (95% CI)	β (95% CI)	β (95% CI)
**Individual factors**				
Daily LPA time	0.09 (0.07, 0.11)^***^	0.09 (0.07, 0.11)^***^	0.09 (0.06, 0.12)^***^	0.08 (0.05, 0.11)^***^
Sex^1^	−4.8 (−7.8, −1.8)^***^	−3.9 (−6.6, −1.2)^***^	−5.9 (−10.6, −1.3)^**^	0.4 (−3.8, 4.7)
Maternal education				
Low	Ref	–	Ref	–
Medium	1.7 (–1.2, 4.6)	–	1.6 (−2.9, 6.1)	–
High	−2.5 (−5.5, 0.6)*	−2.1 (−4.5, 0.4)*	2.4 (−2.2, 7.0)	–
School year	−2.4 (−5.4, 0.6)	–	−3.8 (−8.2, 0.6)*	−4.2 (−7.6, −0.8)^**^
BMI Category				
Normal weight	Ref	–	Ref	–
Overweight	2.9 (−1.0, 6.8)	–	2.6 (−3.3, 8.4)	–
Barriers to PA	0.5 (−2.1, 3.1)	–	−1.8 (−4.4, 1.7)	–
**Behavioural factors**				
Sport/PA during recess	3.1 (−0.1, 6.2)*	−0.9 (−2.9, 2.6)	6.3 (1.7, 10.9)^***^	7.5 (2.1, 12.9)^***^
Bring sports equipment	3.0 (−1.8, 7.8)	–	10.8 (4.5, 17.0)^***^	7.2 (0.5, 13.9)^**^
Borrow sports equipment	1.5 (−3.2, 6.2)	–	2.6 (−3.9, 9.0)	–
**Social factors**				
Peer support	1.8 (−3.1, 6.6)	–	1.0 (−3.8, 5.9)	–
Family support	−0.9 (−3.1, 1.3)	–	−0.7 (−4.3, 2.9)	–
**Organisational/policy factors**				
Break time duration	−0.2 (−0.4, 0.1)	–	0.1 (−0.3, 0.4)	–
**Total variance explained**		38%		41.5%

* *p*<0.1, ^**^ *p*<0.05, ^***^ *p*<0.01.

–  =  Not entered in fully-adjusted model.

1 Males are the referent group.

a Separate models for each dependent variable.

b Adjusted for all significant variables from the crude model.

c Adjusted for T2 LPA and all significant variables from the crude model.

**Table 5:** Cross-sectional and longitudinal associations between individual, behavioural, social and organisational/policy factors and break time%MVPA.

Variables	Cross-sectional		Longitudinal	
	Crude model^a^	Fully-adjusted model^b^	Crude model^a^	Fully-adjusted model^b^
	β (95% CI)	β (95% CI)	β (95% CI)	β (95% CI)
**Individual factors**				
Daily MVPA time	0.17 (0.1, 0.2)^***^	0.15 (0.1, 0.2)^***^	0.22 (0.2, 0.3)^***^	0.20 (0.1, 0.3)^***^
Sex^1^	−3.9 (–5.6, –2.2)^***^	–1.3 (2.8, 0.2)*	–4.6 (–7.4, –1.8)^***^	0.3 (–2.3, 2.9)
Maternal education				
Low	Ref	–	Ref	–
Medium	–0.3 (–2.1, 1.5)	–	1.2 (–1.4, 3.7)	–
High	0.5 (–1.4, 2.3)	–	2.1 (–0.7, 4.8)	–
School year	–2.4 (–4.2, –0.7)^***^	–1.3 [–2.7, 0.1]*	–0.7 (–3.5, 2.1)	–
BMI Category				
Normal weight	Ref	–	Ref	
Overweight	–0.1 (–2.3, 2.4)	–	–0.6 (–4.0, 2.8)	–
Barriers to PA	–0.5 (–2.0, 1.0)	–	0.6 (–1.2, 2.4)	–
**Behavioural factors**				
Sport/PA during recess	4.1 (2.3, 5.8)^***^	0.4 (–1.3, 2.1)	1.9 (–0.4, 4.3)*	4.1 (1.5, 6.7)^***^
Bring sports equipment	4.6 (1.7, 7.4)^***^	3.2 (0.9, 5.4)**	–1.3 (–5.1, 2.6)	–
Borrow sports equipment	5.1 (2.5, 7.8)^***^	1.8 (–0.5, 4.1)	1.9 (–2.2, 6.0)	–
**Social factors**				
Peer support	0.2 (–1.7, 2.0)	–	–1.2 (–4.0, 1.6)	–
Family support	–0.3 (–1.7, 1.1)	–	–0.6 (–2.5, 1.4)	–
**Organisational/policy factors**				
Break time duration	0.1 (0.0, 0.3)*	0.01 (–0.1, 0.1)	0.1 (–0.2, 0.3)	–
**Total variance explained**		34%		45.4%

* *p*<0.1, ^**^ *p*<0.05, ^***^ *p*<0.01.

–  =  Not entered in fully-adjusted model.

1 Males are the referent group.

a Separate models for each dependent variable.

b Adjusted for all significant variables from the crude model.

c Adjusted for T2 MVPA and all significant variables from the crude model.

### Change results

Very few of the correlates examined at T2 were associated with%SED,%LPA and%MVPA two years later. Females continued to engage in more sedentary time during break times than males two years later. Adolescents in higher school year levels also engaged in more sedentary time and less%LPA two years later. Engaging in sport or physical activity during school recess was significantly associated with more%LPA and%MVPA longitudinally. Lastly, adolescents who brought sports equipment to school engaged in more%LPA during break times two years later compared to those who did not bring in equipment to school.

## Discussion

Increasing knowledge concerning correlates of adolescent physical activity and sedentary time is important for health promotion efforts in schools. It is logical to examine cross-sectional and longitudinal associations between different factors and physical activity, as this will provide insights into potential strategies that may be effective in the longer-term if implemented. Moreover, this information has the potential to identify which sub-groups of the population may benefit from physical activity promotion strategies during school break time. Consistent with studies that have objectively [31] and subjectively [10], [32] measured adolescent physical activity in this context, males engaged in more physical activity and less sedentary time than females. There is a need to establish why adolescent males are more active than females to inform future intervention efforts, and future research should be adequately powered in order to examine correlates of their break time physical activity separately. Interestingly, while both males' and females' engagement in%MVPA was low during break time, approximately 40% of their school break time was spent engaged in%LPA. This raises questions whether interventions during break time should focus on increasing%MVPA or increasing overall physical activity participation (i.e., LPA and MVPA), particularly as bringing sports equipment to school was associated with higher %LPA during school break time two years later.

Adolescents' have previously suggested that the provision of sports equipment and organised activities during lunchtime may benefit their physical activity levels [33]. Indeed, the provision of organised activities to adolescents during school break time has been found to be positively associated with MVPA [9]. The present study lends some support to these findings as engaging in sport/physical activity during break time was longitudinally associated with higher%LPA and%MVPA engagement. It is possible that adolescents who choose to engage in sport/physical activity during break time retain this pattern of activity over time. However, this finding also suggests that identifying strategies to enable and sustain participation in these behaviours warrants further attention, particularly as decreases in adolescent break time physical activity have been observed over time [11]. Such strategies may be particularly important for adolescent girls, given their lower physical activity levels in school break time.

Previous research has found that social support from parents, peers and school are correlates of daily [12], [34] and break time [13], [17] physical activity. No cross-sectional or longitudinal associations were found for family and peer social support in this study. The first finding may be explained by their increasing independence from their parents [34]. During this transition, friends become increasingly important as a source of social support and help to establish social norms concerning physical activity behaviours [12], [34]. Previous research has found that the number of active friends adolescents have was associated with daily physical activity [35] and informal game play at school [13], whilst not having any friends to be active with was a barrier to lunchtime physical activity [32]. The present study did not determine the number of friends adolescents had to be active with during break time (a limitation of this study), which may explain, in part, the lack of associations obtained. In addition, the measures used to examine family and peer support may also have impacted on the findings as these were related to support for physical activity overall rather focusing specifically on break time, which is one opportunity for regular engagement in physical activity. Further research is needed to establish the extent to which social support from peers, parents and the school are associated with break time physical activity and sedentary time, and whether strategies for developing social support for activity may be effective for increasing physically active behaviours during school break time.

This present study found a positive cross-sectional association between%MVPA and bringing in sports equipment. Moreover, being allowed to bring in sport equipment to school was also positively associated with%LPA during break time two years later. Adolescents have identified that providing access to school-owned equipment would be one approach that would facilitate their activity choices [33], [36], yet this study suggests that encouraging students to bring in their own sports equipment from home may facilitate physical activity engagement. It is possible that adolescents who can bring in their own sports equipment are more motivated and interested in being physically active [37]. Overall, these findings suggest that permitting adolescents to bring in their own equipment may reduce perceptions of there being nothing to do during school break time [33] and may be simple but effective strategy for increasing physical activity engagement and decreasing sedentary time during break time. However, further research is needed to examine the effectiveness of school policies relating to bringing in equipment on adolescents' physical activity levels as no other studies have examined this association to date [21].

The strengths of this study include objective measurement of physical activity and sedentary time during break times, and the longitudinal data collection. There are, however, several limitations that warrant attention. First, no data were collected concerning the actual behaviours that the adolescents engaged in during break time. Directly observing behaviour, for example, would provide further information concerning how adolescent's use break time to engage in physical activity and sedentary behaviours. In particular, understanding which activities contribute to%LPA engagement during break time is likely to inform intervention strategies, particularly as it accounts for ∼40% of the school day. Second, it is not known whether the adolescents had access to structured lunchtime activities, both sports and academic related, and if so how often they were attended and how long for. It is possible that such lunchtime activities may have impacted on the results obtained. Third, few school level variables were available for analysis in the present study. Correlates such as facility availability, playground size, number of children attending the school, and break time policies, which have been associated with physical activity levels in preschool and elementary school children [19], [38] warrant further attention in adolescents. Fourth, the small sample size for the analyses may affect the generalizability of the results and the ability to determine significant associations, particularly as the magnitude of change in physical activity and sedentary time between T2 and T3 was small.

## Conclusions

A number of individual, behavioural and organisational/policy variables were associated with sedentary time,%LPA and%MVPA during school break time. This study provides an insight into the modifiable variables that could form the focus of interventions in adolescents that target both%LPA and%MVPA engagement. Promising intervention approaches include the provision of equipment and bringing in sports equipment to school. Further research is needed to identify whether enhancing social support for physical activity may also benefit activity levels during school break time.

## References

[1] Andersen LB, Harro M, Sardinha LB, Froberg K, Ekelund U, et al (2006) Physical activity and clustered cardiovascular risk in children: A cross-sectional study (The European Youth Heart Study). Lancet 368: 299–304. 16860699 10.1016/S0140-6736(06)69075-2

[2] Rothon C, Edwards P, Bhui K, Viner RM, Taylor S, et al (2010) Physical activity and depressive symptoms in adolescents: a prospective study. BMC Medicine 8: 32 Available: http://www.biomedcentral.com/1741-7015/8/32 Accessed 20 May 2012. 20509868 10.1186/1741-7015-8-32 PMC2895574

[3] Department of Health (2004) At least five a week: Evidence on the impact of physical activity and its relationship to health. A report from the Chief Medical Officer. London: Department of Health. 128 p.

[4] Department of Health and Ageing (2005) Australia's physical activity recommendations for children and young people 2005. Available:http://www.health.gov.au/internet/main/publishing.nsf/Content/health-pubhlth-strateg-phys-act-guidelines. Accessed 2012 Jun 7.

[5] US Department of Health and Human Services (2008) Physical activity guidelines for Americans. pp 15–20. Available: http://www.health.gov/paguidelines/pdf/paguide.pdf. Accessed 2012 Jun 7.

[6] Commonwealth Scientific Industrial Research Organisation (2008) 2007 Australian National children's nutrition and physical activity survey. Canberra: Commonwealth Scientific Industrial Research Organisation.

[7] Kimm SYS, Glynn NW, Kriska AM, Fitzgerald SL, Aaron DJ, et al (2000) Longitudinal changes in physical activity in a biracial cohort during adolescence. Med Sci Sports Exerc 32: 1445–1454. 10949011 10.1097/00005768-200008000-00013

[8] Ridgers ND, Carter LM, Stratton G, McKenzie TL (2011) Examining children's physical activity and play behaviours during school playtime over time. Health Educ Res 26: 586–595. 21422119 10.1093/her/cyr014

[9] Haug E, Torsheim T, Samdal O (2008) Physical environmental characteristics and individual interests as correlates of physical activity in Norwegian secondary schools: The health behaviour in school-aged children study. Int J Behav Nutr Phys Act 5: 47 Available: http://www.ijbnpa.org/content/5/1/47. Accessed 2012 May 15. 18823545 10.1186/1479-5868-5-47 PMC2564975

[10] Haug E, Torsheim T, Sallis JF, Samdal O (2010) The characteristics of the outdoor school environment associated with physical activity. Health Educ Res 25: 248–256. 18936270 10.1093/her/cyn050 PMC2839138

[11] Ridgers ND, Timperio A, Crawford D, Salmon J (2012) Five-year changes in school recess and lunchtime and the contribution to children's daily physical activity. Br J Sports Med 46: 741–746. 21596716 10.1136/bjsm.2011.084921

[12] Kirby J, Levin KA, Inchley J (2011) Parental and peer influences on physical activity among Scottish adolescents: A longitudinal study. J Phys Act Health 8: 785–793. 21832293 10.1123/jpah.8.6.785

[13] Ommundsen Y, Klasson-Heggebo L, Anderssen SA (2006) Psycho-social and environmental correlates of location-specific physical activity among 9- and 15- year-old Norwegian boys and girls: the European Youth Heart Study. Int J Behav Nutr Phys Act 3: 32 Available: http://www.ijbnpa.org/content/3/32. Accessed 2012 Mar 3. 16999865 10.1186/1479-5868-3-32 PMC1592114

[14] Sallis JF, Owen N (2002) Ecological models of health behaviour. In: Glanz K, Rimer BK, Marcus Lewis F, editors Health Behavior and Health Education: Theory, Research and Practice. 3rd ed. San Francisco: Jossey-Bass. pp. 462–484.

[15] Sallis JF, Prochaska JJ, Taylor WC (2000) A review of correlates of physical activity of children and adolescents. Med Sci Sports Exerc 32: 963–975. 10795788 10.1097/00005768-200005000-00014

[16] van der Horst K, Chin A, Paw MJ, Twisk JWR, van Mechelen W (2007) A brief review on correlates of physical activity and sedentariness in youth. Med Sci Sports Exerc 39: 1241–1250. 17762356 10.1249/mss.0b013e318059bf35

[17] Hohepa M, Scragg R, Schofield G, Kolt GS, Schaaf D (2007) Social support for youth physical activity: Importance of siblings, parents, friends and school support across a segmented school day. Int J Behav Nutr Phys Act 4: 54 Available: http://www.ijbnpa.org/content/4/1/54. Accessed 2012 Feb 16. 17996070 10.1186/1479-5868-4-54 PMC2186357

[18] Haug E, Torsheim T, Samdal O (2010) Local school policies increase physical activity in Norwegian secondary schools. Health Prom Int 25: 63–72. 10.1093/heapro/dap040 PMC2824600 19884244

[19] Ridgers ND, Fairclough SJ, Stratton G (2010) Variables associated with children's physical activity levels during recess: the A-CLASS project. Int J Behav Nutr Phys Act 7: 74 Available: http://www.ijbnpa.org/content/7/1/74. Accessed 2012 Mar 1. 20937142 10.1186/1479-5868-7-74 PMC2959085

[20] Kriemler S, Meyer U, Martin E, van Sluijs EMF, Andersen LB, et al (2011) Effect of school-based interventions on physical activity and fitness in children and adolescents: a review of reviews and systematic update. Br J Sports Med 45: 923–930. 21836176 10.1136/bjsports-2011-090186 PMC3841814

[21] Ridgers ND, Salmon J, Parrish AM, Stanley RM, Okely AD (2012) Physical activity during school recess: A systematic review. Am J Prev Med 43(3): 320–328. 22898126 10.1016/j.amepre.2012.05.019

[22] Timperio A, Salmon J, Ball K, Te Velde SJ, Brug J, et al (2012) Neighborhood characteristics and TV viewing in youth: Nothing to do but watch TV? J Sci Med Sport 15: 122–128. 21955388 10.1016/j.jsams.2011.07.010

[23] Freedson P, Pober D, Janz KF (2005) Calibration of accelerometer output for children. Med Sci Sports Exerc (suppl 11): S523–S530. 10.1249/01.mss.0000185658.28284.ba 16294115

[24] Trost SG, Loprinzi PD, Moore R, Pfeiffer KA (2011) Comparison of accelerometer cut-points for predicting activity intensity in youth. Med Sci Sports Exerc 43: 1360–1368. 21131873 10.1249/MSS.0b013e318206476e

[25] Ridgers ND, Salmon J, Ridley K, O'Connell E, Arundell L, et al (2012) Agreement between activPAL and ActiGraph for assessing children's sedentary time. Int J Behav Nutr Phys Act 9: 15 Available: http://www.ijbnpa.org/content/9/1/15. Accessed 2012 Feb 19. 22340137 10.1186/1479-5868-9-15 PMC3311087

[26] Mo F, Turner M, Krewski D, Mo FD (2005) Physical inactivity and socioeconomic status in Canadian adolescents. Int J Adolesc Med Health 17: 49–56. 15900811

[27] Cole T, Bellizi M, Flegal K, Dietz WH (2000) Establishing a standard definition for child overweight and obesity worldwide: International survey. BMJ 320 1240–1243. 10797032 10.1136/bmj.320.7244.1240 PMC27365

[28] Plewis I, Fielding A (2003) What is multi-level modelling for? A critical response to Gorard. Br Educ Stud 51: 408–419.

[29] Crawford D, Cleland V, Timperio A, Salmon J, Andrianopoulos N, et al (2010) The longitudinal influence of home and neighbourhood environments on children's body mass index and physical activity over 5 years: the CLAN study. Int J Obes 34: 1177–1187. 10.1038/ijo.2010.57 20351728

[30] Tabachnick B, Fidell L (1996) Using multivariate statistics. 3rd ed. New York: Harper Collins. 880 p.

[31] Martinez-Gomez D, Calabro MA, Welk GJ, Marcos A, Veiga OL (2010) Reliability and validity of a school recess physical activity recall in Spanish youth. Pediatr Exerc Sci 22: 218–230. 20567043 10.1123/pes.22.2.218

[32] Hohepa M, Scragg R, Schofield G, Kolt GS, Schaaf D (2009) Self-reported physical activity levels during a segmented school day in a large multiethnic sample of high school students. J Sci Med Sport 12: 284–292. 18328781 10.1016/j.jsams.2007.11.005

[33] Hohepa M, Schofield G, Kolt GS (2006) Physical activity: What do high school students think? J Adolesc Health 39: 328–336. 16919793 10.1016/j.jadohealth.2005.12.024

[34] Mulhall P, Reis J, Bebum S (2011) Early adolescent participation in physical activity: Correlates with individual and family characteristics. J Phys Act Health 8: 244–252. 21415452 10.1123/jpah.8.2.244

[35] Leggett C, Irwin M, Griffith J, Xue L, Fradette K (2012) Factors associated with physical activity among Canadian high school students. Int J Public Health 57: 315–324. 21952906 10.1007/s00038-011-0306-0

[36] Stanley RM, Boshoff K, Dollman J (2012) Voices in the playground: A qualitative exploration of the barriers and facilitators of lunchtime play. J Sci Med Sport 15: 44–51. 21940211 10.1016/j.jsams.2011.08.002

[37] Wilson DK, Williams J, Evans A, Mixon G, Rheaume C (2005) Brief report: a qualitative study of gender preferences and motivational factors for physical activity in underserved adolescents. J Pediatr Psychol 30: 293–297. 15784925 10.1093/jpepsy/jsi039 PMC2830817

[38] Cardon G, Van Cauwenberghe E, Labarque V, Haerens L, De Bourdeaudhuij I (2008) The contribution of preschool playgaygound factors in explaining children's physical activity during recess. Int J Behav Nutr Phys Act 5: 11 Available: http://www.ijbnpa.org/content/5/1/11. Accessed 2012 Oct 10. 18302741 10.1186/1479-5868-5-11 PMC2266778

